# Practical guide for managing large-scale human genome data in research

**DOI:** 10.1038/s10038-020-00862-1

**Published:** 2020-10-23

**Authors:** Tomoya Tanjo, Yosuke Kawai, Katsushi Tokunaga, Osamu Ogasawara, Masao Nagasaki

**Affiliations:** 1 National Institute of Informatics, Tokyo, 101-8430 Japan; 2 Genome Medical Science Project, National Center for Global Health and Medicine, Tokyo, 162-8655 Japan; 3 The Bioinformation and DDBJ Center, National Institute of Genetics, Mishima, Shizuoka, 411-8540 Japan; 4 Center for the Promotion of Interdisciplinary Education and Research, Kyoto University, Sakyo-ku, Kyoto 606-8507 Japan; 5 Center for Genomic Medicine, Kyoto University Graduate School of Medicine, Sakyo-ku, Kyoto 606-8507 Japan

**Keywords:** Biotechnology, Genetics research

## Abstract

Studies in human genetics deal with a plethora of human genome sequencing data that are generated from specimens as well as available on public domains. With the development of various bioinformatics applications, maintaining the productivity of research, managing human genome data, and analyzing downstream data is essential. This review aims to guide struggling researchers to process and analyze these large-scale genomic data to extract relevant information for improved downstream analyses. Here, we discuss worldwide human genome projects that could be integrated into any data for improved analysis. Obtaining human whole-genome sequencing data from both data stores and processes is costly; therefore, we focus on the development of data format and software that manipulate whole-genome sequencing. Once the sequencing is complete and its format and data processing tools are selected, a computational platform is required. For the platform, we describe a multi-cloud strategy that balances between cost, performance, and customizability. A good quality published research relies on data reproducibility to ensure quality results, reusability for applications to other datasets, as well as scalability for the future increase of datasets. To solve these, we describe several key technologies developed in computer science, including workflow engine. We also discuss the ethical guidelines inevitable for human genomic data analysis that differ from model organisms. Finally, the future ideal perspective of data processing and analysis is summarized.

## Introduction

In human genetics, advancements in next-generation sequencing technology have enabled population-scale sequencing from just one sequencer and allowed sharing millions of human genome sequencing data from publicly archived data including privacy-protected ones. With the development of various bioinformatics tools, maintaining the productivity of research, managing human genome data, and analyzing downstream data is essential. This review aims to guide researchers in human genetics to process and analyze these large-scale genomic data to extract relevant information for improved downstream analyses in their specific research domains.

Here, in each section, we answer the five inevitable questions for human genome data processing and analysis: (i) what kind of large-scale human genome projects are underway and available from data sharing? (ii) how to store and analyze human genome data efficiently? (iii) what kind of computational platforms are used to store and analyze human genome data? (iv) how to maintain reproducibility, portability, and scalability in genome data analysis, and why is it important? (v) which policy should be followed to handle human genome data?

In “What kind of large-scale human genome projects are underway and available from data sharing?” section, we inform large-scale human genomic studies in worldwide and how the data produced in these studies are sharing. Lots of effort and cost are inevitable for storing and processing the human genomic data obtained by whole-genome sequencing (WGS). Therefore, in “How to store and analyze human genome data efficiently?” section, we focus on the development of data format and software that manipulate WGS including hardware-based acceleration.

Once the sequencing is complete and its format and data processing tools are ready, a computational platform must be selected, as discussed in “What kind of computational platforms are used to store and analyze human genome data?” section. For the platform, we recommend a multi-cloud strategy for balancing cost, performance, and customizability. A high-quality published research relies on data reproducibility to ensure quality results, reusability for applications to other datasets, as well as scalability for the future increase of datasets. “How to maintain reproducibility, portability, and scalability in genome data analysis, and why is it important?” section describes the method to solve these demands using several key technologies, such as container technology, workflow description languages, and workflow engines. The ethical guidelines inevitable for human genomic data analysis that differ from model organisms are discussed in “Which policy should be followed to handle human genome data?” section. Finally, the future ideal perspective of human genome data processing and analysis in human genetics are discussed.

## What kind of large-scale human genome projects are underway and available from data sharing?

Several early collaborative large-scale human genome analyses have been conducted worldwide. The Human Genome Project (HGP) [1] is one of the largest and most successful international collaborations in genome science. Researchers in institutes throughout the world contributed to sequence all the bases in the human genome and assembled them to construct one human reference assembly, followed by attempts to catalog genes hidden in the human reference assembly. The assembly is the major achievement of HGP, and the reference genome data is freely accessible from a very early phase. The Genome Reference Consortium (GRC) has taken over updating and maintaining the assembly of human reference genome, and the updated versions of the human genome assembly are commonly used by researchers around the world. Nowadays, all researchers depend on the coordinate of the human reference assembly from GRC. Therefore, the HGP study initially exemplified the importance of data sharing in genome science.

After the great success of HGP, the human genome study has shifted toward studying the diversity of human genomes. The HapMap Project [2] was one of the early large-scale population genomics studies used to systematically analyze individual genotypes on a population scale. In this project, single nucleotide polymorphisms (SNPs) representing human genetic diversity were discovered and genotyped using SNP genotyping array technology, which was popular at the time. In phase 1 study, the project completed genome-wide genotyping of 269 individuals from 4 populations. Finally, the analysis was extended to 1184 individuals from 11 populations in phase 3 study. This was the first study that revealed the structure of linkage disequilibrium in human genome across the populations. The International 1000 Genomes Project is a successor to the HapMap project. This study aimed to comprehensively elucidate the genetic diversity of human populations by utilizing next-generation sequencers, which was being put to practical use at the time. In phase 1 study, whole genomes of 1092 individuals from 14 populations were sequenced by next-generation sequencers. The analysis eventually expanded to 2,504 individuals from 26 populations in phase 3 study [3], and then continued to incorporate new technologies, such as 3rd generation long leads sequencers [4]. Importantly, all data and derivatives from the above-mentioned genome studies are available with open access data sharing policy.

Therefore, these data are not only used as summary statistics, e.g., a catalog of allele frequencies of SNPs in populations, but also used as individual-level information, e.g., a whole-genome reference panel, which contains individual genotype information for whole-genome regions, especially useful for genotype imputation to SNP genotyping arrays, e.g., Japonica Array [5]. Open access policy also has the advantage of being used by many researchers. The data from the International 1000 Genomes Project has contributed to the development of a variety of NGS tools. Currently, common tools for NGS data analysis, e.g., bwa [6] and *de-facto* standard formats, e.g., Sequence Alignment/Map (SAM), BAM, and VCF [7], have been developed in the International 1000 Genomes Project. In addition, the genomic data are widely distributed under the open access policy though various computational platforms, e.g., high-performance computing (HPC) system of the National Institute of Genetics (NIG) in Japan and public cloud services. These efforts also ease the reusability by researchers.

Several present large-scale human genome analyses have shifted toward understanding the relationship between genotypes and phenotypes, e.g., diseases and traits. Of these, cohort studies with biobanking play a key role, and many of these are prospective cohort studies of residents of a specific region or country (Table 1) [8–28]. The DNA materials in the biobank allow us to measure the status of the entire genome sequence, e.g., SNP genotyping array or WGS, under the informed consent of participants. The genomic information and phenotypes collected in the cohort study have enabled the large-scale association studies between genotypes and phenotypes. Compared with the former International 1000 Genomes Project, trait information for participants is available, and many studies have shared their individual genomic data under controlled access to protect the individual’s privacy. Notably, varying policies to data sharing for controlled access have an impact on collaborative studies across regions or countries. UK Biobank with nearly 500,000 participants distributes their data (including individual genomic data) to the approved research studies, and these distributed data can be analyzed on the computational platform of each study group while ensuring security. Instead, many studies have not adopted the flexible data sharing policy like UK Biobank and currently hinder the reusability and collaboration of researchers. Sharing the summary statistics is still the predominant method in international collaborations, and many of the GWAS meta-analyses have been successful in this way.　However, there are still barriers to sharing data at the individual level, which hinders collaborative research that requires advanced analysis. Discussions on how to share data in a flexible manner while protecting individual privacy should continue to take place. One promising direction might be the recently proposed cloud-based solution from UK Biobank (https://www.ukbiobank.ac.uk/2020/08/uk-biobank-creates-cloud-based-health-data-analysis-platform-to-unleash-the-imaginations-of-the-worlds-best-scientific-minds/)

**Table 1:** Large-scale cohort studies with genomic information

Project	Description	Website	Country	Reference
Human Genome Project (HGP)	The Initial sequencing program of the human genome	https://www.genome.gov/human-genome-project	International	[1]
International HapMap Project	Study of the common pattern of human genetic variation using SNP array	https://www.genome.gov/10001688/international-hapmap-project	International	[2]
1000 Genomes Project	Determining the human genetic variation by means of whole-genome sequencing in population scale	https://www.internationalgenome.org	International	[3]
Human Genome Diversity Project	Biological samples and genetic data collection from different population groups throughout the world	https://www.hagsc.org/hgdp/	International	[8]
Simon Genome Diversity Project	Whole-genome sequencing project of diverse human populations	https://docs.cancergenomicscloud.org/v1.0/docs/simons-genome-diversity-project-sgdp-dataset	International	[9]
Genome Asia 100k	WGS-based genome study of people in South and East Asia	https://genomeasia100k.org/	International	[10]
UK Biobank	Biobank study involving 500,000 residents in the UK	https://www.ukbiobank.ac.uk	UK	[11]
Genomics England	WGS-based genome study of patient with rare disease and their families and cancer patients in England	https://www.genomicsengland.co.uk/	UK	[12]
FinnGen	Nationwide biobank and genome cohort study in Finland	https://www.finngen.fi/en	Finnland	[13]
Tohoku Medical Megabank Project	Biobank and genome cohort study for local area (north-east region) in Japan	https://www.megabank.tohoku.ac.jp/english	Japan	[14, 15]
Biobank Japan	Nationwide patient-based biobank and genome cohort study in Japan	https://biobankjp.org/english/index.html	Japan	[16]
Trans-Omics for Precision Medicine (TOPMed)	A genomic medicine research project to perform omics analysis pre-existing cohort samples	https://www.nhlbiwgs.org	USA	[17]
BioMe Biobank	Electronic health record-linked biobank of patients from the Mount Sinai Healthcare System	https://icahn.mssm.edu/research/ipm/programs/biome-biobank	USA	[18]
Michigan Genomics Initiative	Electronic health record-linked biobank of patients from the University of Michigan Health System	https://precisionhealth.umich.edu/our-research/michigangenomics/	USA	[19]
BioVU	Repository of DNA samples and genetic information in Vanderbilt University Medical Center	https://victr.vumc.org/biovu-description/	USA	[20]
DiscovEHR	Electronic health record-linked genome study of participants in Geisinger’s MyCode Community Health Initiative	http://www.discovehrshare.com/	USA	[21]
eMERGE	Consortium of biorepositories with electronic medical record systems and genomic information	https://www.genome.gov/Funded-Programs-Projects/Electronic-Medical-Records-and-Genomics-Network-eMERGE	USA	[22]
Kaiser Permanente Research Bank	Nationwide biobank collecting genetic information from a blood sample, medical record information, and survey data on lifestyle from seven areas of US	https://researchbank.kaiserpermanente.org/	USA	[23]
Million Veteran Program	Genome cohort study and biobank of participants of the Department of Veterans Affairs (VA) health care system	https://www.research.va.gov/mvp/	USA	[24]
CARTaGENE	Biobank study of 43,000 Québec residents	https://www.cartagene.qc.ca/en/home	Canada	[25]
lifelines	Multigenerational cohort study that includes over 167,000 participants from the northern population of the Netherlands	https://www.lifelines.nl/	Netherlands	[26]
Taiwan Biobank	Nationwide biobank and genome cohort study of residents in Taiwan	https://www.twbiobank.org.tw/test_en/index.php	Taiwan	[27]
China Kadoorie Biobank	Genome cohort study of patients with chronic diseases in China	https://www.ckbiobank.org/site/	China	[28]

## How to store and analyze human genome data efficiently?

The sequencing data once generated, must be stored in a specific format. In the past, various sequencing formats have been proposed, e.g., CSFASTA/QUAL format. Fortunately, the current *de-facto* standard is the fastq format, which is a text-based format with sequencing bases and the quality score for each base (base quality score, BQS). The definitions of BQS range are different among vendors and/or versions, e.g., the quality score ranges from 33 (corresponds to! in the ascii code table) to 73 (I) in Sanger format and 64 (@) to 104 (h) in the Solexa format. In the early days of NGS technology, problems due to variations were not speculated. The Sequencing Read Archive (SRA) in the National Center for Biotechnology Information (NCBI) is responsible for storing raw sequencing data in the United States (US). For reusability for users, normalized data of the BQS (quality adjusted to the standardized format) are also stored and distributed from SRA. The data are now shifting toward public clouds, i.e., Google Cloud Platform (GCP) and Amazon Web Service (AWS). Users have no end-user charges for accessing cloud SRA data in the cloud, whether in hot or cold storage when the user is accessing the data from the same cloud region (more details in https://www.ncbi.nlm.nih.gov/sra/docs/sra-cloud-access-costs/).

The process of storing original sequencing data and handling BQS in the sequenced reads to reduce sequencing data size in clouds are being studied (https://grants.nih.gov/grants/guide/notice-files/NOT-OD-20-108.html). The total size in SRA was 36 petabytes in 2019 and major parts were consumed by the BQS. A solution is to downsample the BQS by binning. Without the BQS, the size of a typical SRA file reduces by 60–70%. Thus, another extreme opinion is to remove the BQS for the standard dataset in clouds.

The sequenced data with the fastq format is typically aligned to the human reference assembly, usually Genome Reference Consortium Human Build 38 (GRCh38) or GRCh37, by using bioinformatics tools, such as bwa [6] and bowtie2 [29]. The *de-facto* standard output format is the SAM text format, which stores each fastq read with the chromosomal position and the alignment status, e.g., mismatches to the bases in the reference sequences to the reference coordinate (if a fastq read is not located in the reference, the fastq read is stored in the unmapped section) (https://samtools.github.io/hts-specs/SAMv1.pdf). Commonly, this text format was stored as the BGZF compression format (extended format from a standard gzip fromat), called BAM format (https://samtools.github.io/hts-specs/SAMv1.pdf). Recently, the European Bioinformatics Institute (EBI) proposed the reference sequence, e.g., GRCh37 and GRCh38, based compression format called CRAM [30]. Contrary to BAM, the CRAM has two compression schemes, lossless or lossy format, i.e., downsample the BQS and offering 40–50% space saving over the alternative BAM format with the lossless option (http://www.htslib.org/benchmarks/CRAM.html). The dataset for the International 1000 Genomes Project can be downloaded in the BAM format (in total 56.4 terabyte for low-coverage phase 3 dataset aligned to GRCh37 reference assembly) as well as lossy CRAM format with 8-bin compression scheme to reduce the total download size (https://www.internationalgenome.org/category/cram/) compared to the BAM format (in total 18.2 terabyte for the same dataset aligned to GRCh38DH reference assembly).

The aligned sequenced data are then called variants by tools to detect variants, e.g., Genome Analysis ToolKit (GATK) [31, 32] and Google’s DeepVariant for germline variant call [33] and MuTect2 for somatic variant call [34].

The alignment and variant call for thousands of WGS dataset require adequate computational resources. Therefore, to reduce the computation time for these WGS analyses, several hardware or software-based solutions have been proposed [35]. The NVIDIA Clara™ Parabricks developed the Graphics Processing Unit (GPU)-accelerated tools (https://www.parabricks.com/). The Illumina DRAGEN™ Platform uses highly reconfigurable Field-Programmable Gate Array technology (FPGA) to provide the other hardware-accelerated implementations of genomic analysis algorithms [36]. The Sentieon analysis pipelines implement software-based optimization algorithms and boost the calculation performance compared with the native tools, such as GATK and MuTect2 [37]. These platforms are available both on the on-premises and on the public clouds. The storage cost in public cloud for clinical sequence has been discussed by Krumm et al. [38].

## What kind of computational platforms are used to store and analyze human genome data?

For effective genome data sharing and analysis, not only the security and legal compliance issues should be addressed, but also researchers need to deal with the recent data explosions and be familiar with the large-scale computational and networking infrastructures.

As a solution that addresses both issues, commercial cloud platforms have been gaining attention recently. The world-leading cloud platforms, e.g., GCP, AWS, and Microsoft Azure, are achieving and maintaining compliance with complex regulatory requirements, frameworks, and guidelines. This does not mean that the organization providing some services on the cloud platforms will be automatically certified under those regulations; however, utilizing cloud platforms can make it easier for researchers to meet the compliance [39–43].

In addition to the privacy compliance issue, as a consequence of recent data explosion in GWAS and NGS research [44], copying data to the researcher’s on-premise servers has become increasingly difficult since projects utilizing thousands of genomes need to operate on several hundred terabytes of data, which could take months to download. Therefore, for large-scale data analysis, data visiting strategy has emerged as a realistic solution where instead of bringing data to researchers, the researchers operate on the data where it resides, e.g., data of International 1000 Genomes Project are stored on AWS and NIG as described. The data visiting strategy can be implemented naturally on commercial cloud platforms.

Broad Institute provides a GWAS and NGS data analysis pipeline execution environment called Terra on GCP [45]. Terra allows researchers to execute many analysis workflows on the workflow engine called Cromwell, and it also offers a workflow reuse and exchange environment for research reproducibility, without taking the ownership of the computational infrastructure and its management. Terra and Cromwell on the GCP are one of the best starting points for middle-scale data analysis projects.

In addition, since the distributed nature of the cloud is especially efficient for large collaborative projects, many NGS research projects, in particular, the reanalysis of large-scale archived datasets and large genomics collaborations funded by the US agents, are utilizing the cloud computing platforms as their primary computational infrastructures [46, 47].

Especially, NCBI in National Institutes of Health (NIH) is now trying to move the computational infrastructure of the comprehensive DNA database toward commercial cloud platforms. The International Nucleotide Sequence Database Collaboration (INSDC) that operates among The DNA Data Bank of Japan (DDBJ), The European Bioinformatics Institute (EMBL-EBI), and NCBI has been developing comprehensive DNA sequence databases via DRA, ERA, and SRA in each region. NCBI is moving SRA data on the GCP and AWS platforms (each about 14PB; https://ncbiinsights.ncbi.nlm.nih.gov/2020/02/24/sra-cloud/) as part of the NIH Science and Technology Research Infrastructure for Discovery, Experimentation, and Sustainability (STRIDES) Initiative [48].

On the other hand, cloud computing also has some intrinsic real-world problems, such as vendor-lock in, unpredictable cost of computing, networking and data storage, inconsistent security, and multiple management tools.

According to the investigation in July 2018 [49, 50], >80% of companies around the world describe their cloud strategy as multi-cloud, commonly defined as using multiple public and private clouds for different application workloads. In general, multi-cloud strategy is used for making balances between costs, performances, and customizability. Again, it poses challenges to provide consistent infrastructure and easy operations across multiple-cloud vendors and on-premise computers. Several cutting-edge computer technologies can be used for these purposes, as described later, especially the Linux container technologies and its federation on some dedicated management middleware including Virtual Cloud Provider (VCP) developed by the National Institute of Information (NII) [51], Kubernetes (https://kubernetes.io/), and Apache Mesos (http://mesos.apache.org/).

In the INSDC, Europe and Japan can be classified into the multi-cloud strategy. Computational infrastructure, which supports the analysis and development of these huge databases, is also massive. In the DDBJ, the NIG supercomputer system is offered to medical and biological researchers who require large-scale genome data analysis. The current system (which started operation in 2019) is equipped with about 14,000 cores CPUs with the peak performance of 1.1 PFLOPS (CPU: 599.8 TFLOPS, GPU 499.2 TFLOPS); the total storage capacity is 43.8 petabyte, and each component is interconnected with high-speed network (100 Gbps InfiniBand) suitable for large-scale data analysis [52].

The NIG supercomputer provides 16 GPU nodes that allow genome analysis tools, including GATK [32] and Mutect2 [34], to accelerate more than one order, by using a dedicated analysis system, e.g., Parabricks genome pipeline. It also offers large-scale shared memory (12 terabyte in total) computer mainly used for *de novo* genome assembly [52].

The security and legal compliance for the personal genome analysis environment of the NIG supercomputer is supervised by the National Bioscience Database Center (NBDC) in the Japan Science and Technology Agency (JST), and the NIG supercomputer is designated as available server outside of the affiliated organization (“Off-premise-server”) in Japan (https://humandbs.biosciencedbc.jp/en/off-premise-server). The system is connected to the commercial cloud vendors including AWS via the SINET5 network system hosted by NII, Japan [53], and on this platform, we have developed a multi-cloud infrastructure with the cooperation among National Institute of Information, Hokkaido University, Tokyo Institute of Technology, Kyushu University, and National Institute of Genetics.

## How to maintain reproducibility, portability, and scalability in genome data analysis, and why is it important?

Reproducibility of the data analysis results is one of the main concerns in the biomedical field [51, 54] since the version of applications and configuration to applications affect the results. To maintain the reproducibility of the experimental results, in publication, it has become common to describe each data processing, e.g., version of tools and configuration to tools, steps of these data processing, and dataset used in the data analysis (e.g., sequencing data and phenotypes). These descriptions allow researchers to re-construct workflows (also known as pipelines), consisting of a sequence of data analysis applications, in their laboratories. There are several solutions to denote workflows. The naive workflows are constructed with bare programming languages, e.g., Java or Python, or software build systems, e.g., GNU make (https://www.gnu.org/software/make/) or SCons (https://scons.org/). Usually, researchers deploy the applications by downloading and/or building the source codes by themselves. However, this naive workflow sometimes causes several limitations. First, deploying applications to every computing resource is difficult because of library dependencies, including system libraries, as well as the versions of compilers or interpreters are to be considered. If the tool still deploys, the libraries of different versions might affect the result of data analysis. Second, efficiently executing workflows on different computing resources is difficult because the computational node of data processing is sometimes hard-coded in the programming language. For example, a workflow written in GNU make cannot utilize several computing nodes simultaneously, except for combination to batch job systems, because the tool supports the parallel execution solely in a single computational node. In modern data analysis, researchers can solve these limitations by combining key technologies in computer science, the container technology, workflow description languages, and workflow engines (also known as Scientific Workflow Management Systems (SWfMS) or Workflow Management Systems (WMS)). The container technology allows deploying the same tools including its library dependencies to different computational platforms while preserving the computational performance. Workflow description languages and workflow engines enable researchers to separate the description of workflow and the physical computational platform that processes the workflow.

## Containers

Containers have been commonly used to publish applications [55] as well as provide an isolated computing environment, e.g., virtual machine [56]. Although several container engines are proposed (https://opencontainers.org/) [57, 58], essential concepts for users are the same: a container image, a container runtime, and a container registry (many literatures omit the words “image,” “runtime,” and “instance” (explained later) and simply call them “container”), as described below. First, a container image, e.g., Docker image, OCI image (a variant of Docker image) (https://opencontainers.org/), and SIF image [57], is a package that contains all the dependencies including system libraries to execute the application. Each container image is identified by its container image ID, e.g., “6b362a9f73eb,” or the pair of container name and its tag name, e.g., “docker/whalesay:latest” (“docker/whalesay” is the container name and “latest” is the tag name). A container image can be built from the script named “Dockerfile” (for Docker images) or Singularity definition file (for SIF images). Users can build almost the same container image from a given script. Note that only providing the script file is not enough to build completely the same container image because the script can refer to external resources. The strictest solution to use the same container image is to refer to the unique image by specifying the same container ID that is already published in the container registry, which is explained later. Second, a container runtime, e.g., Docker engine [59] and Singularity [57], is a system to execute tools in a given container image. An executed process using a container image is called a container instance. A container runtime provides an isolated file system using the container image to the container instance as if it is dedicated to the host. By using resource isolation features, e.g., namespace in Linux kernel, rather than hardware emulation, e.g., virtual machines, a container runtime can execute a container instance as efficiently as the host process [60]. In the bioinformatics field, Docker engine and Singularity are widely used for data analysis applications. Docker engine is a container runtime that is widely used for building data analysis environments [51, 61] as well as for building educational applications [62]. It supports Docker images and OCI images. Although it required root privileges for any container manipulations in older versions, it experimentally supports executing container images in user privileges since version 19.03. Singularity is another container runtime, especially for HPC fields. It supports SIF images as well as Docker images. It only requires user privileges and therefore some HPC systems have better support for Singularity, e.g., NIG [52]. Finally, a container registry, e.g., DockerHub (https://hub.docker.com/), Quay.io (https://quay.io/), and SingularityHub (https://singularity-hub.org/), is a repository that stores and publishes container images. Container images built by other registry users can be downloaded from a container registry; researchers can publish their container images in the container registry. However, when using container images built by other registry users, it is important to verify that they do not contain security vulnerabilities. Fortunately, some images are already verified by the container registry provider or by the community. For example, DockerHub provides verified images for well-known Linux distributions, programming language environments, and tools. Another example is BioContainers [55], which provides bioinformatics applications that are verified by the BioContainers community. Other types of container images can be verified by checking the script such as “Dockerfile” or using security scanning tools for containers such as Docker-Bench-for-Security (https://github.com/docker/docker-bench-security), Clair (https://github.com/quay/clair), and Trivy (https://github.com/aquasecurity/trivy).

## Workflow engines, workflow description languages, and their ecosystems

A workflow engine is a system to execute workflows and can encapsulate how a given workflow is controlled and executed, e.g., the decision of the order of executions of applications and the re-execution of the failed execution steps, and how a given workflow is executed on the different computing resources where a given workflow is executed (e.g., cloud computing resources and computing nodes in batch job schedulers). Using a workflow engine, users can execute workflows on various computing resources without changing workflow definitions. A workflow description language describes applications and workflow definitions for workflow engines. A tool description includes input parameters, output parameters, a container image for execution, and an execution command, whereas a workflow description includes connections between applications and workflows. By using workflow description languages, users can construct workflows without taking care of the execution details of workflows such as how and where workflows are executed.

However, it is difficult for users to choose appropriate workflow engines from existing 280+ workflow engines (https://github.com/common-workflow-language/common-workflow-language/wiki/Existing-Workflow-systems) that satisfy each demand. Users have to convert workflow definitions to port it to other workflow engines manually in general because each workflow engine supports only one or a few workflow description languages; as described later, using the Common Workflow Language (CWL) or the Workflow Description Language (WDL) is a good choice to keep portability between workflow engines. Furthermore, they have differences in the supported computing resources, ecosystems, e.g., workflow editors, visualizers, reusable tools, and workflow repositories. To help users choose appropriate workflow engines, we briefly introduce several workflow engines and workflow description languages, including their ecosystems. For more details, see [63] and literature for each engine and language.

The Galaxy [64] is a workflow manager with a web user interface and enables users to execute workflows without using a command-line interface. It also provides a GUI workflow editor, tool repository, execution history of workflows, and many other features. It has been mainly developed by Penn State University and Johns Hopkins University since 2005. Although users can build their own Galaxy server, there is another choice to use public Galaxy servers that service commonly used applications and reference genomes (https://galaxyproject.org/use/). We can learn how to use Galaxy from official training materials (https://training.galaxyproject.org/training-material/).

**Fig. 1:**
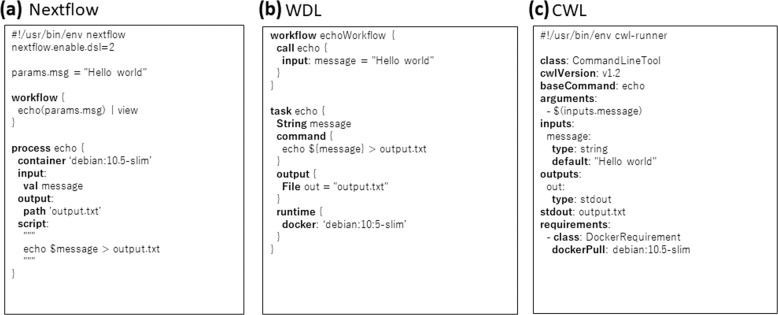
The simple hello world example by using workflow description languages: (**a**) Nextflow, (**b**) WDL, and (**c**) CWL

The Nextflow [65] is another workflow engine as well as a domain-specific language (DSL). Nextflow has a Groovy-based DSL, as shown in Fig. 1a, and is easy to understand if users are already familiar with some programming languages. Nextflow also has the GUI frontend [66]. It has been developed by the Comparative Bioinformatics group at the Barcelona Centre for Genomic Regulation (CRG) since 2013. A curated set of tool and workflow descriptions can be found at nf-core [56] and DockStore [67].

The WDL (https://openwdl.org/) is a community-driven specification and is supported by several workflow engines, e.g., Cromwell (https://github.com/broadinstitute/cromwell), MiniWDL (https://github.com/chanzuckerberg/miniwdl), and dxWDL (https://github.com/dnanexus/dxWDL). WDL was first developed by the Broad Institute and is currently developed by the OpenWDL community (see Fig. 1b). It has been officially supported on Terra platform by Broad Institute [45]. We can find a curated set of tool and workflow descriptions at BioWDL (https://github.com/biowdl) and DockStore [67]. Note that this paper uses the words “WDL” and capitalized “Workflow Description Language” to indicate the language by OpenWDL community but some literatures use the same words to indicate a language to describe workflows.

**Fig. 2:**
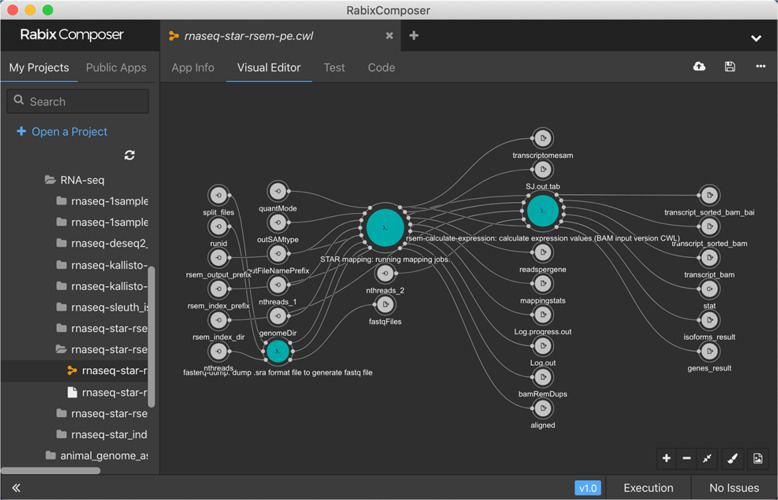
Example of the GUI editor of workflow engine; snapshot of the Radix Composer. The flow shows an RNA-Seq pipeline

The CWL (https://w3id.org/cwl/v1.2/) is another community-driven specification and has superior portability between workflow engines. It has been supported by over 14 workflow engines, including alpha stage (https://www.commonwl.org/#Implementations). Although the YAML-based syntax (see Fig. 1c) makes it difficult to understand, there have been many systems that assist to read/write tool and workflow definitions, e.g., GUI editor like Rabix Composer (https://rabix.io/) (see Fig. 2) and converters from/to other languages (https://www.commonwl.org/#Converters_and_code_generators). A curated set of tool and workflow descriptions can be found at Common Workflow Library (https://github.com/common-workflow-library) and DockStore [67].

## Advantages of using modern data analysis compared with traditional approaches

By switching from naive workflow to the modern workflow, users can obtain reproducibility, portability, and scalability for large-scale data analysis. First, the container technology allows reproducibility of the published results. In a naive workflow, when an application is installed on the HPC system by an administrator, then the administrator is responsible for proper working of the application. When the application is installed on the user’s computational environment, then the person who installed it, usually, the user, has the responsibility. However, it is sometimes impossible to install the same version of application on user’s environment that is installed on HPC systems due to version conflicts between several HPC systems, for example. Conversely, in the case of modern workflow, the maintainer of the corresponding container images for the application has the responsibility. Therefore, a user can use the same application between HPC systems and his or her computational environment by using the same container image. Second, the combination of a workflow description language and workflow engines allows the portability to different computational environments and the scalability of data analysis that adapts to the increase of the size of computational resources. Naive workflows are described in programming languages or build tools. Therefore, it is nearly impossible to execute workflows on different types of computing resources without modifying the workflow description. In the modern workflow, the difference in computing resources is encapsulated by the workflow engines. For example, a workflow description once written in CWL can be executed on local machines, computing resources on cloud platforms, and computing nodes of batch job schedulers. Terra and Cromwell on the GCP are one of the solutions for scalability with a modern approach. Notably, to work modern workflows on multi-platforms, the administrator of each platform needs to properly install container runtimes, e.g., docker engine and singularity.

## Instruction to write a workflow using CWL

**Fig. 3:**
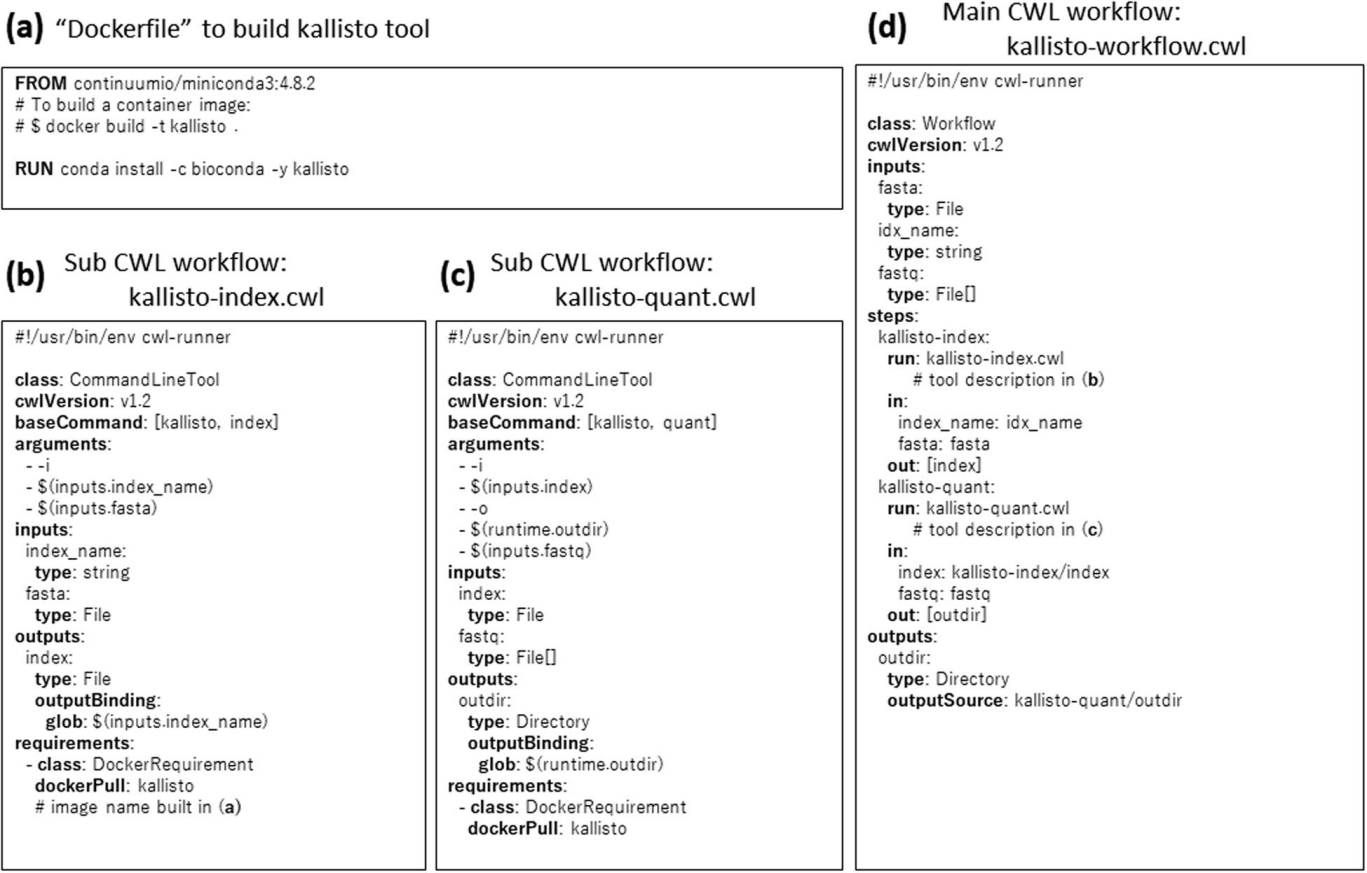
Example of “Dockerfile”, a tool description, and a workflow description of kallisto workflow in CWL. **a** “Dockerfile” to build an RNA-Seq fastq data processing tool kallisto. **b** A CWL sub-workflow used in **d**. The workflow creates the index file for kallisto of the target transcript sequences with fasta format. **c** CWL sub-workflow used in **d**. The workflow processes RNA-Seq fastq files to generate their abundance of transcripts. **d** The main CWL workflow operates the sub-workflow in **b** and **c**

This section shows an example of how to write a workflow that uses Docker engine in CWL. We can apply the similar idea for other container runtime and workflow description languages. Here, the workflow in Fig. 3 implements RNA-Seq data processing operations; (i) the workflow takes three inputs; a fasta file with target transcript sequences, the name of generated index, and list of RNA-Seq files with fastq format; (ii) kallisto [68] indexes the fasta file; (iii) kallisto processes the list of fastq files and generates the transcript abundance information.

There are three steps to write a workflow: containerize tools, write tool descriptions, and write a workflow description. Before applying each step, check that a containerized tool, a tool description, or a workflow description is not published by the community. If the workflow is already published, we recommend using the published one.

First, we search for an appropriate base image for the tool to be containerized. For example, using “continuumio/miniconda3:4.8.2” published in DockerHub can be an appropriate image for tools in Bioconda. Note that base images with the “latest” tag are not appropriate to maintain reproducibility because its contents vary when the new version is released. Once we choose a base image, we can write a “Dockerfile” to extend a base image. In the simplest case, it can be done by using the “FROM” instruction to specify the base image and the “RUN” instruction to specify the installation commands as shown in Fig. 3a. For more details of “Dockerfile”, see the official document (https://docs.docker.com/engine/reference/builder/).

Second, we write a tool description for each tool in the workflow. As shown in Fig. 3b, c, it specifies the list of input parameters (“inputs” field), output parameters (“outputs” field), a container name (“dockerPull” field), and how the execution command is constructed (“baseCommand” and “arguments” fields). In Fig. 3b, “$(inputs.index_name)” and “$(inputs.fasta)” in the “arguments” fields are instantiated by the values of “index_name” and “fasta” parameters, respectively. A file name of “index” parameter in the “outputs” field is captured by using the value of “index_name” parameter.

In Fig. 3c, “$(runtime.outdir)” in the “arguments” and “glob” fields is instantiated by the output directory name and therefore the “outdir” parameter in the “outputs” field captures the directory that contains all the output files of the “kallisto quant” command.

Finally, we can write a workflow description by referring to tool descriptions as shown Fig. 3d. In the case of CWL, we refer to the external tool definition in the “run” field and refer to the output parameters in other steps by using “other-step/output-parameter” notation. A workflow engine can recognize the dependencies of input and output parameters for each step and therefore it can execute the ‘kallisto-quant‘ step before executing the ‘kallisto-index‘ step without specifying the order of executions.

## Which policy should be followed to handle human genome data?

In general, personal data protection law has two closely related aims: (a) protection of privacy and security during the data processing and (b) establish acceptable rules for data transfer across societies or countries [69]. The transborder restriction is necessary to prevent the data protections from being circumvented by simply moving the data to the country of other jurisdictions [39]. At the same time, the transborder restriction rule must find the balance between the protection of privacy and the benefits of data sharing that affects a variety of activities including science and commerce [39]. Under this background, the European Union (EU)’s General Data Protection Regulation (GDPR) came into force in May 2018 as the successor of the EU Data Protection Directive (1995) (https://gdpr-info.eu/) [40]. The GDPR facilitates the free movement of data among the Member States of the EU, and transferring personal data to a country outside the EU is allowed only when one of the conditions laid out in Chapter V of the GDPR is fulfilled. These include the following: (a) The destination has been the subject of an adequacy decision, (b) Binding corporate rules (BCRs), and (c) Standard data protection clauses (SDPC) (https://gdpr-info.eu/) [40]. Japan and the EU agreed to recognize each other’s data protection regimes as providing adequate protections for personal data in July 2018, and the framework for mutual and smooth transfer of personal data between Japan and European Union came into force on 23 January 2019 [40, 70, 71]. In the Japanese regime, “Act on the Protection of Personal Information” (http://www.japaneselawtranslation.go.jp/law/detail/?id=2781&vm=2&re=02) is one of the central parts of the personal data protection regime [70], and under this framework, “Cabinet Order to Enforce the Act on the Protection of Personal Information” prescribes personal genome data as a kind of an individual identification code. Associated with this law, and in order to facilitate computerization and data sharing [72, 73], three ministries published two security guidelines, where the first guideline is for a medical institution, and the other guideline is for companies operating on healthcare information:

1. Security Guidelines for Medical Information Systems, 5th Edition (May, 2017). Ministry of Health, Labor and Welfare (MHLW).
2. Guidelines for Safety Management of Medical Information by Providers of Information Systems and Services Handling Medical Information (August, 2020), the Ministry of Internal Affairs and Communications (MIC) and the Ministry of Economy, Trade and Industry (METI).

The “Security Guidelines for Medical Information Systems” describes technical details of security countermeasures that should be considered in medical institutes, organizational management, physical security, human resources security, computer and network security, disaster recovery, information lifecycle management, and consideration on data exchange. In addition to the technical details, this guideline also determines responsibility division points between ICT users (i.e., medical institutions) and ICT providers. Here, it should be noted that the text “Chapter IV: Obligations etc. of a Personal Information Handling Business Operator” of the “Act on the Protection of Personal Information” governs only the private organizations of Japan. Government or public sector organizations of Japan are not subject to Chapter IV. They are governed by other series of laws including “Act on the Protection of Personal Information Held by Administrative Organs,” “Act on General Rules for Incorporated Administrative Agency,” and bylaws of local public organizations. Consequently, the GDPR adequacy decision to Japan is implied to be limited to the private sector of Japan, and the government and the public sectors need data transfer with subject to appropriate safeguards (e.g., Art.46 GDPR). In the US, the following laws govern the healthcare and genome data sharing operations: the Federal Policy for the Protection of Human Subjects (known as the “Common Rule”), the Health Insurance Portability and Accountability Act (HIPAA), Health Information Technology for Economic and Clinical Health Act (HITECH), and Health Information Trust Alliance Common Security Framework (HITRUST CSF) [74]. However, the US lacks federal data privacy law, and the above US laws governing health care data sharing do not impose different requirements on transborder data sharing, even if it is transferred to third countries, compared with data sharing among researchers or service providers inside the US [74]. Consequently, the European Commission cannot grant the US an adequacy decision, and it is worth noting that transfer personal data needs to be subjected to appropriate safeguards, e.g., Art.46 GDPR. To remedy this situation, a data transfer mechanism called the EU–US Privacy Shield was adopted by the European Commission in July 2016 and became available on August 1, 2016 [41]. However, we need to be cautious with the unstable situation. On July 16, 2020, the Court of Justice of the European Union issued a judgment declaring as “invalid” on the adequacy of the protection provided by the EU-U.S. Privacy Shield (https://www.privacyshield.gov/Program-Overview).

## Conclusion and future direction

Twenty years have passed since the release of human reference genome assembly. With the advancement of the sequencing technology, hundreds and thousands of whole-genome sequencing can be obtained in single institute within a short period. In addition, WGS data analysis applications, including hardware and software-based solutions, would accelerate to allow large-scale data analysis on multi-cloud by integrating their dataset to available human genome data with population scale via their data sharing policy. The data analyses would also be built on modern workflow engines and easily ensure the reproducibility of publication. The workflows on publications are also shared in the research community. With the portability, the pipeline would be reused in other dataset on different computational environments. The pipeline would also be scaled to a large dataset with the functionality of scalability.

Therefore, in human genetics, from the outputs of the workflow engines to the large-scale human genome data, more domain-specific downstream data interpretations would be demanded from both the expert-knowledge driven approach by the domain knowledge from the medical and biological professionals and the data-driven approach from computer science, e.g., artificial intelligence.
